# Comparison of the Technical Success of Obtura and StarClose Systems for Femoral Arterial Access-Site Closure in Neurointerventional Procedures: A Single-Center Retrospective Cohort Study

**DOI:** 10.3390/jcm15218242

**Published:** 2026-10-23

**Authors:** Nurcan Kat, Tirdad Setayeshi

**Affiliations:** Department of Radiology, Bursa Yuksek Ihtisas Training and Research Hospital, University of Health Sciences, 16310 Bursa, Türkiye; tirdadtf@gmail.com

**Keywords:** vascular closure device, hemostasis, femoral artery, neurointerventional procedures, manual compression

## Abstract

**Background/Objectives:** To compare technical hemostasis outcomes of collagen-based Obtura and nitinol clip-based StarClose vascular closure devices (VCDs) for femoral access-site closure after transfemoral neurointerventional procedures in two cohorts treated during non-overlapping periods. **Methods:** In this single-center retrospective cohort study, 166 of 250 consecutive patients undergoing transfemoral neurointervention (2022–2025) were included after exclusions; closure was performed via a 6F sheath with Obtura in 2025 (*n* = 98) or StarClose in 2022–2024 (*n* = 68). The primary endpoint was hemostasis with gentle pressure within 2 min of device deployment without additional procedure or prolonged manual compression. Unadjusted, adjusted, and propensity-score-matched (PSM) analyses were performed, along with a threshold-sensitivity analysis. **Results:** Technical success was 52.9% in the StarClose period and 70.4% in the Obtura period (OR, 2.11; 95% CI, 1.11–4.03; *p* = 0.033); the association was not significant after adjustment (OR, 1.99; 95% CI, 0.99–3.99; *p* = 0.053) or PSM (OR, 1.55; 95% CI, 0.72–3.30; *p* = 0.261). Additional compression time among failures was shorter in the StarClose period (5.5 vs. 10.0 min; *p* < 0.001). One pseudoaneurysm and one hematoma occurred per group; no major complications occurred. A ≤7-min threshold reversed the direction (OR, 0.77; 95% CI, 0.38–1.57; *p* = 0.592). **Conclusions:** The devices were used in completely different, non-overlapping periods; therefore, device effects cannot be separated from period effects. These findings do not demonstrate the superiority of either device. They are hypothesis-generating and provide a basis for designing prospective, concurrent comparative studies.

## 1. Introduction

Neurointerventional procedures are increasingly performed as a result of technological advances and the expanding use of evidence-based applications. Because a substantial proportion of these procedures are performed through the femoral artery, achieving safe and effective hemostasis at the access site after the procedure is important for procedural success and patient safety. Control of the femoral arteriotomy has become particularly important in patients receiving anticoagulant or antiplatelet therapy [1,2].

Manual compression has long been used as the reference method for achieving hemostasis after femoral arterial access, and vascular closure devices (VCDs) have primarily been compared with this method in clinical studies [3]. However, the need for prolonged compression may extend postprocedural immobilization and reduce patient comfort [4,5]. Vascular closure devices were developed to reduce these limitations of manual compression and have been compared with manual compression in randomized studies as an alternative method for achieving hemostasis at the femoral access site [3,6].

VCDs achieve hemostasis through different mechanisms. Obtura™ (Meril Life Sciences, Vapi (Gujarat), India) is a mechanical closure system in which an intravascular anchor and an extravascular collagen sponge support the arteriotomy from both sides. Hemostasis is achieved by compressing the arteriotomy between these two components, while the thrombogenic properties of collagen may also facilitate the hemostatic process [7]. StarClose (Abbott Vascular, Redwood City, California, USA) is a nitinol clip-based closure system. The device achieves hemostasis by externally approximating the arterial wall using a nitinol clip placed extravascularly at the arteriotomy site. A key feature of the device is that no permanent closure material is left within the vascular lumen [8].

Previous literature includes studies comparing Obtura with manual compression [7] and data regarding the use of StarClose in neurointerventional applications [2]. However, data directly comparing these two devices, which have different closure mechanisms, within the same neurointerventional clinical context remain limited.

The aim of this study was to compare the technical hemostasis success of the Obtura and StarClose systems used for femoral arterial access-site closure following transfemoral neurointerventional procedures and to evaluate the duration of additional manual compression required in cases of technical failure. Because the two devices were used in consecutive, non-overlapping periods at our center, the findings should be interpreted as observed outcome differences between these periods rather than as evidence of the superiority of one device over the other.

## 2. Materials and Methods

### 2.1. Study Design and Ethics

This was a single-center, retrospective cohort study comparing two consecutive, non-concurrent cohorts, based on a retrospective review of the clinical records of patients who underwent neurointerventional procedures via transfemoral access at the Department of Radiology, Bursa Yuksek Ihtisas Training and Research Hospital, University of Health Sciences, between 2022 and 2025.

The study protocol was approved by the Medical Sciences Ethics Committee of Bursa Yuksek Ihtisas Training and Research Hospital, University of Health Sciences (protocol no.: 2024-TBEK 2026/08-12). Because of the retrospective nature of the study, a waiver of the requirement for informed consent was granted. All procedures were performed in accordance with the principles of the Declaration of Helsinki.

### 2.2. Patient Population

A total of 250 consecutive patients who underwent neurointerventional procedures via transfemoral access during the specified period and whose arteriotomy was closed with Obtura Obtura™ (Meril Life Sciences, Vapi (Gujarat), India) or StarClose (Abbott Vascular, Redwood City, CA, USA) were screened. Eighty-four patients meeting at least one predefined exclusion criterion were excluded, and 166 patients were included in the final analysis. Sixty-eight patients were in the StarClose period and 98 were in the Obtura period.

Inclusion criteria were age 18–90 years, use of transfemoral arterial access, completion of a 30-day postprocedural color Doppler ultrasonography examination, closure of the postprocedural arteriotomy with Obtura or StarClose, and use of only a 6 French (6F) femoral procedural sheath in both groups.

Exclusion criteria were use of manual compression or a VCD other than the study devices, sheath diameter other than 6F, diagnostic angiography alone, more than one access attempt during the same session, femoral arterial anatomy unsuitable for device deployment, active infection at the femoral access site, and incomplete data.

Femoral arterial anatomy was considered unsuitable for device use in the presence of severe calcification, significant stenosis, a small vessel diameter (<5 mm), or a puncture site located above the inguinal ligament or below the femoral bifurcation.

Because the approved indication for StarClose is limited to 5–6F procedural sheaths, only patients in whom a 6F sheath was used were included in order to minimize sheath diameter as an important confounder.

### 2.3. Device Selection and Procedural Protocol

Device selection was not based on patient or lesion characteristics; rather, it was determined by which device was available at the center during the relevant period. StarClose was used from January 2022 through December 2024, whereas Obtura was used from January 2025 through December 2025. Thus, device type and period of use were completely non-overlapping in this study. Consequently, device effects and period effects cannot be statistically separated. This was recognized a priori as a major methodological limitation of the study and was taken into account in interpretation of the results.

In both periods, all procedures were performed by the same interventional radiology team and by the same interventional radiologist with 15 years of experience; no new operators joined during the study period. According to the protocol of the Department of Interventional Radiology, the femoral puncture technique did not change, and the same 6F sheath system was used throughout the study. Although this provided a degree of consistency in technical application, the overlap of operator, period, and device within the same time intervals was not considered an independent advantage attributable to the device.

Procedures were performed under general anesthesia or sedation according to the type of neurointerventional procedure and patient characteristics. Systemic heparinization was administered with a target activated clotting time (ACT) of 250–300 s during the procedure. The heparinization protocol did not change during the study; in both periods, systemic heparin was administered with the same target ACT of 250–300 s. Heparin reversal with protamine was not part of the study protocol. Antiplatelet and anticoagulant treatments were determined according to procedural indications. Patients who received intravenous thrombolytic therapy before or during mechanical thrombectomy were not included during the study period.

Both VCDs were deployed according to the manufacturers’ instructions for use. Additional manual compression was applied in cases of unsuccessful device deployment. After the procedure, all patients underwent standardized 4-h bed rest and limb immobilization. Data on early ambulation and patient comfort were not systematically available in the retrospective records and therefore were not evaluated in the study.

At 30 days after the procedure, all patients underwent color Doppler ultrasonography for assessment of femoral access-site complications, regardless of symptoms. Doppler examinations were performed by an experienced radiologist who was not a member of the interventional radiology team and who was unaware of the VCD used at the time of assessment.

### 2.4. Data Collection

The following variables were recorded from patient files: age, sex, diabetes mellitus, hypertension, smoking status, antithrombotic medication use, procedure type, VCD used, intraprocedural heparin dose, device success, duration of additional manual compression required in cases of failure, and access-site complications.

Procedures were categorized into four groups: (1) aneurysm embolization, (2) carotid/intracranial stenting, (3) mechanical thrombectomy, and (4) other procedures.

### 2.5. Outcomes

Primary endpoint: The primary endpoint was defined as cessation of arterial bleeding from the groin access site after deployment of the vascular closure device without additional manual compression or additional intervention, other than gentle manual pressure for a maximum of 2 min. This interval was retrospectively calculated as the difference between the recorded time of device deployment and the recorded time of cessation of bleeding.

At our center, when hemostasis is not achieved immediately after deployment of a vascular closure device, gentle manual pressure for up to 2 min is applied as part of routine clinical practice, regardless of device type. Therefore, instead of the 3-min threshold used for StarClose in previous studies, a 2-min threshold reflecting the routine clinical practice of our center was chosen. The same threshold was applied equally to both devices to ensure that technical hemostasis success was assessed using a common criterion.

Previous studies have used a 3-min additional-compression threshold for StarClose and a 2-min threshold for another VCD [9,10].

Technical failure was defined as failure to successfully deploy the VCD despite appropriate anatomical conditions and a technique consistent with the manufacturer’s recommendations, failure to achieve adequate hemostasis despite device application, or the need for additional manual compression to achieve hemostasis.

Secondary endpoint: The secondary endpoint was the duration of additional manual compression applied to achieve hemostasis following technical failure.

Major bleeding was defined, adapted from the Bleeding Academic Research Consortium (BARC) criteria, as bleeding requiring transfusion, administration of prothrombin complex concentrate, or surgical/endovascular repair [11].

### 2.6. Statistical Analysis

Continuous variables were presented as mean ± standard deviation (SD) or median (interquartile range [IQR]), according to distribution, and categorical variables were presented as number and percentage. The Mann–Whitney U test was used for between-group comparisons of continuous variables, and Fisher’s exact test was used for categorical variables. Standardized mean differences (SMDs) were calculated to assess baseline covariate balance, and |SMD| > 0.10 was considered indicative of potential imbalance. Results of logistic regression models are presented as odds ratios (ORs) with 95% confidence intervals (CIs) calculated using the Wald method.

To reduce baseline differences between device groups, the propensity score (PS) was estimated using a logistic regression model with device group as the dependent variable. The model included age, sex, diabetes, hypertension, systolic blood pressure, aspirin use, clopidogrel use, enoxaparin use, procedure type (three categories; the single “other” procedure was included in the stenting group), and intraprocedural heparin dose. Because sheath diameter was 6F and intraprocedural heparin was used in 100% of patients, these variables were not included in the model. Patients were matched 1:1 without replacement using nearest-neighbor matching in random order (primary analysis, random seed 42) with a caliper equal to 0.2 times the standard deviation of the logit of the PS. Covariate balance was reassessed using SMDs after matching. Technical success in the matched cohort was compared between device groups using McNemar’s exact test. The device effect in the matched cohort was estimated using conditional logistic regression; as a sensitivity analysis, covariates with residual imbalance (|SMD| > 0.10) were added to this model.

To identify factors independently associated with technical success in the full cohort, a multivariable logistic regression model was constructed. The model included device group, age, sex, aspirin use, a composite antithrombotic burden score, and a mechanical thrombectomy indicator. The composite antithrombotic burden score was calculated as the sum of aspirin, clopidogrel, and enoxaparin use, ranging from 0 to 3. The appropriateness of modeling the score as a continuous linear variable was evaluated by comparing continuous and categorical (0, 1, ≥2) models using a likelihood-ratio test. A sensitivity analysis excluding aspirin from the model was performed to assess potential collinearity because aspirin was a component of the composite score. A potential interaction between device group and antithrombotic burden score was explored by adding an interaction term. Model performance was assessed using the events-per-variable (EPV) ratio, the Hosmer–Lemeshow goodness-of-fit test, and the c-statistic. Firth penalized logistic regression was also performed to reduce bias related to the small sample size and sparse events. Exploratory analyses included subgroup analyses by procedure type (mechanical thrombectomy vs. other procedures), with a device × mechanical thrombectomy interaction term, and by antithrombotic burden; technical success by calendar year within the StarClose period (Cochran–Armitage test for trend); and the mean additional compression time per treated patient, counting 0 min for patients with technical success.

Sensitivity analyses were performed to assess the robustness of the findings using alternative definitions of technical success (total manual-compression thresholds of ≤3, ≤5, and ≤7 min, with crude and multivariable-adjusted estimates), the effect of outliers, sensitivity of matching to different random orderings, and sensitivity to unmeasured confounding. The E-value was used to assess unmeasured confounding. Because the outcome prevalence was not low, odds ratios were converted to risk ratios (RRs) before calculation of the E-value using the square-root approximation (RR ≈ √OR). Because device and period were completely collinear, it was recognized that E-value analysis cannot eliminate the structural confounding resulting from the complete overlap between device use and procedural period.

All tests were two-sided, and *p* < 0.05 was considered the threshold for statistical significance. Analyses were performed using Python v3.12 (Python Software Foundation License) and R v4.3.3 (Foundation for Statistical Computing, Vienna, Austria).

## 3. Results

### 3.1. Study Population

A total of 250 patients who underwent neurointerventional procedures through transfemoral arterial access were screened. After application of the predefined exclusion criteria, 84 patients were excluded and 166 patients were included in the final analysis. Among the excluded patients, 28 had manual compression and/or a sheath other than 6F, 37 underwent diagnostic angiography only, 13 had missing data, and 6 had unsuitable femoral access. The study included 68 patients in the StarClose period and 98 patients in the Obtura period (Figure 1).

### 3.2. Baseline Characteristics

Baseline characteristics of the groups are presented in Table 1. Age and sex distribution were similar between the groups; however, aspirin use (53.1% vs. 33.8%; *p* = 0.017) and the composite antithrombotic burden score (median, 1 [IQR, 0–2] vs. 0 [IQR, 0–1]; *p* = 0.011) were significantly higher in the Obtura period. Although clopidogrel use and systolic blood pressure did not reach statistical significance, a total of nine covariates, including these two, exceeded the |SMD| > 0.10 threshold (Table 1). Because of this multidimensional imbalance, propensity-score matching and multivariable adjustment were used in subsequent analyses.

No significant difference was observed between the periods in the distribution of procedure types (Fisher’s exact test, *p* = 0.285) (Table 2).

### 3.3. Primary Endpoint

Technical hemostasis success, the primary endpoint, was 36/68 (52.9%) in the StarClose period and 69/98 (70.4%) in the Obtura period. The crude odds of success were higher in the Obtura period than in the StarClose period (OR, 2.11; 95% CI, 1.11–4.03; *p* = 0.033). In the multivariable model, the direction of the device association was maintained but remained just above the threshold for statistical significance (OR, 1.99; 95% CI, 0.99–3.99; *p* = 0.053). In Firth penalized logistic regression, the direction of the device association was similar (OR, 1.93; 95% CI, 0.97–3.86; *p* = 0.061). Mechanical thrombectomy was associated with lower odds of technical hemostasis success in the model (OR, 0.23; 95% CI, 0.07–0.77; *p* = 0.017). Age, female sex, aspirin use, and the composite antithrombotic burden score were not significantly associated with technical success (Table 3). The interaction between device and the composite antithrombotic burden score was not statistically significant (*p* = 0.054), modeling the score as a continuous variable was appropriate (likelihood-ratio test, *p* = 0.83), and excluding aspirin from the model did not change the device estimate (OR, 1.99; 95% CI, 0.99–4.00).

In exploratory subgroup analyses, technical success in the StarClose and Obtura periods was 49.0% vs. 59.3% in the mechanical thrombectomy subgroup (OR, 1.52; 95% CI, 0.71–3.26) and 63.2% vs. 87.2% in the non-thrombectomy subgroup (OR, 3.97; 95% CI, 1.06–14.89); the device × mechanical thrombectomy interaction was not significant (*p* = 0.22) (Supplementary Table S1). Within the StarClose period, technical success was 8/18 (44.4%) in 2022, 14/25 (56.0%) in 2023, and 14/25 (56.0%) in 2024 (Cochran–Armitage test for trend, *p* = 0.48; adjusted OR per year, 1.45; 95% CI, 0.74–2.83). The standard intraprocedural heparin dose of 5000 IU was administered to 88.2% of patients in the StarClose period and 95.9% in the Obtura period (*p* = 0.073); adding heparin dose to the multivariable model did not change the device estimate (OR, 1.99; 95% CI, 0.99–4.01).

### 3.4. Propensity-Score Matching

After propensity-score matching, 55 StarClose-period patients were matched 1:1 with 55 Obtura-period patients (Table 4). Baseline imbalances in aspirin and clopidogrel use and in diastolic blood pressure were reduced after matching (diastolic blood pressure SMD = 0.08); residual imbalance at |SMD| > 0.10 remained for sex (−0.15), smoking (−0.15), clopidogrel use (0.12), and enoxaparin use (0.11); 2 vs. 1 patients). Technical success was 30/55 (54.5%) and 36/55 (65.5%), respectively. In the matched cohort, there were 17 discordant pairs in which StarClose failed and Obtura succeeded and 11 pairs in which StarClose succeeded and Obtura failed; McNemar’s exact test showed no statistically significant difference (*p* = 0.345), and the matched conditional OR was 55 (95% CI, 0.72–3.30; *p* = 0.261). In conditional logistic regression, additional adjustment for diastolic blood pressure (OR, 1.51; 95% CI, 0.70–3.25) or for sex, smoking, and clopidogrel use (OR, 1.38; 95% CI, 0.60–3.18) did not materially change the matched estimate. When matching was repeated using 10 different random orderings, OR values ranged from 1.55 to 2.29 and statistical significance was not reached in any repetition. Thirteen StarClose patients and 43 Obtura patients could not be matched because they fell outside the caliper. The technical success rate among unmatched Obtura patients (76.7%) was higher than that among matched Obtura patients (65.5%), limiting the generalizability of the matched analysis to the entire Obtura cohort. Matching balanced the measured baseline characteristics but could not eliminate differences between the periods in which the two devices were used.

### 3.5. Threshold Sensitivity Analysis

Results of the threshold sensitivity analysis are summarized in Table 5. At the total ≤3-min threshold, no patient was reclassified and the result remained unchanged. At the total ≤5-min threshold, one patient in each period was reclassified as successful, and the direction and magnitude of the association were largely preserved. In contrast, at the total ≤7-min threshold, 16 patients in the StarClose period and only 1 patient in the Obtura period were reclassified as successful, reversing the direction of the association. Thus, the magnitude and direction of the observed association between the devices were substantially sensitive to the definition of hemostasis used. These findings were similar in multivariable-adjusted analyses (≤5 min: adjusted OR, 1.98; 95% CI, 0.99–3.96; ≤7 min: adjusted OR, 0.64; 95% CI, 0.29–1.39).

### 3.6. Additional Manual Compression

The duration of additional manual compression was evaluated in 61 technically unsuccessful patients and was significantly shorter in the StarClose period (Mann–Whitney U test, *p* = 4.8 × 10^−7^) (Table 6). Exclusion of outliers did not change the result (*p* = 1.3 × 10^−7^), indicating that the difference was not driven by a single extreme observation. Because compression times were obtained from retrospective medical records, these values should not be interpreted as real-time, prospectively standardized hemostasis times. When all patients were included, counting 0 min for those with technical success, the mean additional compression time was 3.1 min in the StarClose period and 3.3 min in the Obtura period (*p* = 0.37).

### 3.7. Vascular Complications

A pseudoaneurysm and a hematoma occurred in one patient each in both groups, with no significant difference in rates between the groups (Table 7). No major bleeding, transfusion, or bleeding requiring surgical/endovascular repair was observed in the overall cohort. Because of the low number of events, no definitive conclusion regarding a safety difference or equivalence between the two devices was made. At the patient level, any access-site complication occurred in 2/68 (2.9%) patients in the StarClose period and 1/98 (1.0%) in the Obtura period; in the Obtura period, the pseudoaneurysm and hematoma occurred in the same patient.

## 4. Discussion

The most important issue in interpreting this study is the complete non-overlap between device type and period of use. StarClose was used only during 2022–2024, whereas Obtura was used only during 2025. Therefore, device effects cannot be separated from period effects. Changes in operator experience, potential differences in patient selection, changes in procedural protocols, and developments in equipment or technical practice may have contributed to part of the observed difference. This structural confounding cannot be completely eliminated by propensity-score matching or multivariable regression. Therefore, no inference regarding the superiority of either device should be drawn from the results of this study; its main value lies in guiding the design of future concurrent comparative studies. To investigate a possible learning or period effect within the StarClose period, we examined technical success by calendar year. There was no significant trend across the StarClose years (Cochran–Armitage test for trend, *p* = 0.48; adjusted OR per year, 1.45; 95% CI, 0.74–2.83). However, because the annual subgroups were small (18–25 patients), a gradual effect of increasing operator experience cannot be completely excluded.

In this study, the technical hemostasis outcomes of the Obtura and StarClose systems used for femoral arterial access-site closure following transfemoral neurointerventional procedures were compared. In the crude analysis, the technical success rate was higher in the Obtura period than in the StarClose period (70.4% vs. 52.9%; OR, 2.11; 95% CI, 1.11–4.03; *p* = 0.033). After multivariable adjustment, the association was attenuated and was no longer statistically significant (OR, 1.99; 95% CI, 0.99–3.99; *p* = 0.053). A similar direction but more uncertain result was obtained after propensity-score matching (OR, 1. 55; 95% CI, 0.72–3.30; *p* = 0.261). These findings suggest that the difference observed in the crude analysis may have been influenced by baseline differences between the groups and by other measured or unmeasured factors. Accordingly, our results should not be interpreted as definitive evidence that Obtura is superior to StarClose, but rather as a finding requiring confirmation in adequately powered prospective studies in which the devices are compared during the same time period.

When these findings are considered in the context of device-specific literature, high technical success rates have been reported for StarClose in cardiovascular and neurointerventional applications [2,8]. In contrast, the technical success rate in the StarClose period of our study was lower than that observed in the Obtura period. Restricting the study to cases using only 6F sheaths was intended to reduce heterogeneity related to sheath diameter; however, technical success may be affected by various patient-, procedure-, and device-related factors beyond sheath diameter. The literature on Obtura also generally reports high technical success rates. In the randomized study by Devasia et al., Obtura was reported to provide higher technical success and shorter hemostasis time than manual compression [7]; high success rates were also reported in studies by Himanshu et al. and Padhi et al. [12,13]. The technical success rate observed for Obtura in our study was lower than that reported in these series. However, because the populations, procedure types, and definitions of technical success differed from those in our study, direct comparisons of success rates should be made cautiously.

Comparison with head-to-head and device-specific VCD studies places our findings in a broader context (Table 8). In the randomized ISAR-CLOSURE trial of 4524 patients undergoing 6F coronary angiography, device failure occurred in 5.3% with FemoSeal and 12.2% with ExoSeal, and 30-day access-site complications in 6.0% and 7.8%, respectively [14]. In a prospective comparison of 400 patients undergoing percutaneous coronary intervention (PCI) predominantly with 6F sheaths, StarClose could not be successfully deployed or failed to achieve adequate closure in 16 patients, who were excluded from the outcome analysis [15]. A meta-analysis of nine studies (5466 patients) found slightly higher closure success with Angio-Seal than with StarClose (RR, 1.05; 95% CI, 1.00–1.09) and no significant difference in complications [16], and a retrospective study of 166 patients undergoing complex endovascular procedures under intensive anticoagulation reported 100% deployment success with both devices and no major complications [17]. In a four-arm randomized trial, technical success was 99.1% with Angio-Seal, 97.4% with Obtura, and 94.6% with ProGlide, with hematoma rates of 0.8–3.2% and pseudoaneurysm rates of 0–0.8% [12]. In the only neurointerventional series of Obtura that we identified (100 patients, 8F device), hemostasis success was 96% and hematoma or pseudoaneurysm occurred in 6% [13]. The technical success rates observed in both periods of our study were lower than those reported in these studies, whereas access-site complication rates (2.9% in the StarClose period and 1.0% in the Obtura period) were within or below the reported ranges. This difference may be explained in part by our stricter primary endpoint, which allowed no more than 2 min of gentle manual pressure, compared with definitions permitting up to 3 min of compression or based on deployment success alone; by our neurointerventional population, in which most patients underwent emergency mechanical thrombectomy under systemic heparinization; and by the need to exchange the long sheath for a short introducer sheath before closure. Consistent with this interpretation, success in the StarClose period increased to 76.5% when a total compression threshold of ≤7 min was applied (Table 5). Because populations and definitions differ across studies, and because the devices in our study were used in non-overlapping periods, these comparisons are descriptive, and absolute rates should not be compared directly.

The manual-compression threshold used to define the primary endpoint is also important for interpretation of the results. In this study, ≤2 min of mild compression did not invalidate technical success. This threshold was partly consistent with short compression durations used in some previous studies [9,10]. More importantly, the direction of the results changed in the threshold sensitivity analysis. At the ≤5-min total threshold, the direction of the association was unchanged, whereas at the ≤7-min threshold, the direction of the association reversed, and no statistically significant difference was observed between the groups at this threshold (adjusted OR, 0.64; 95% CI, 0.29–1.39). This is an important methodological finding of the study. It highlights the importance of defining hemostasis success in a standardized and prespecified manner in VCD comparative studies.

A substantial proportion of StarClose failures were managed with short additional manual compression, whereas Obtura failures tended to require longer compression. This made the outcome sensitive to the selected threshold value. Therefore, future studies may benefit from evaluating multiple endpoints simultaneously, including successful device deployment, the need for additional compression, and total hemostasis time, rather than relying on a single compression-duration cutoff.

Indeed, in our study, the median duration of additional compression in unsuccessful cases was 5.5 min for StarClose and 10.0 min for Obtura, and this difference persisted after exclusion of outliers. Published data suggest rapid hemostasis after successful Obtura deployment [7,13], whereas tract leakage after StarClose has been reported to require only short manual compression [9,18]. In our center, a 6F long sheath system is frequently used for neurovascular interventions, and both vascular closure systems are designed to operate through a short (11-cm) introducer sheath; therefore, replacement of the long sheath with the short introducer sheath was required at the end of the procedure. Unlike systems supplied with their own dedicated sheath, the Obtura system was not always observed to be compatible with the standard introducer sheaths available at our center. In deep and steeply angled femoral artery access sites, the T-fastener component of Obtura could not advance sufficiently through the sheath opening, resulting in device failure. This observation is presented as a hypothesis-generating explanation that sheath–device incompatibility may have contributed to failures in the Obtura period; sheath–device compatibility was not systematically assessed or tested in this retrospective cohort.

Whether these device characteristics can inform device selection in specific clinical scenarios requires caution. In our cohort, the two characteristics appeared to offset each other: Obtura failed less often but required longer compression when it failed, whereas StarClose failed more often but was usually rescued with brief compression. As a result, the mean additional compression time per treated patient was similar between periods (3.1 min with StarClose vs. 3.3 min with Obtura; *p* = 0.37). In exploratory analyses (Supplementary Table S2), the difference in success between periods was smaller in emergency mechanical thrombectomy (49.0% vs. 59.3%) than in elective carotid/intracranial stenting (53.3% vs. 90.9%) and in patients with a composite antithrombotic burden score ≥2 (56.2% vs. 86.5%); however, these subgroups largely overlapped, were small, were not adjusted for confounders, and the corresponding interaction tests were not significant. Taken together, and given the non-concurrent use of the two devices, our data do not support device-specific recommendations for particular clinical scenarios. They may, however, inform hypotheses for prospective evaluation: for example, whether a device with high immediate success is preferable in patients on dual antiplatelet therapy, in whom prolonged rescue compression may be undesirable, or whether a device that can be rescued with brief compression is preferable in emergency thrombectomy, in which failures are more frequent. Some practical considerations apply irrespective of device choice. In centers using long sheaths for neurointervention, compatibility between the closure device and the short introducer sheath should be confirmed before closure, given our observation regarding the Obtura T-fastener; and, particularly after thrombectomy under systemic heparinization, checking the ACT before closure and preparing for rescue compression may be advisable. Because all patients in our cohort underwent VCD closure, none of these observations can be extended to comparisons with manual compression. Device selection should therefore remain individualized according to femoral anatomy, sheath compatibility, antithrombotic therapy, and operator experience.

Vascular complications were rare in both groups. One pseudoaneurysm and one hematoma occurred in each group. These rates may be broadly consistent with ranges reported in previous series [6,7,16,19]; however, because of the low number of events, no conclusion regarding safety equivalence or superiority between the two devices can be drawn.

Mechanical thrombectomy was associated with lower odds of technical success in the multivariable model (OR, 0.23; 95% CI, 0.07–0.77; *p* = 0.017). However, this association should not be interpreted causally. Unmeasured factors, including the clinical characteristics of patients undergoing mechanical thrombectomy in the setting of acute ischemic stroke, procedure duration, antithrombotic management, and variability in ACT levels, may have contributed to this association. Because sheath diameter was 6F in all patients, the finding cannot be explained by a larger sheath diameter. Nevertheless, because of the observational nature of the study, a direct causal relationship between mechanical thrombectomy and technical hemostasis failure cannot be inferred. Mechanical thrombectomy was associated with lower technical success with both devices compared with other procedures (StarClose, 49.0% vs. 63.2%; Obtura, 59.3% vs. 87.2%), and no significant interaction with device type was observed (*p* = 0.22); however, the subgroup analyses were underpowered to detect effect modification.

One strength of the study was that all patients underwent color Doppler ultrasonography at 30 days after the procedure regardless of symptoms. Doppler examinations were performed by an experienced radiologist who was not part of the interventional radiology team and was unaware of the VCD used at the time of assessment. This approach increases the likelihood of detecting clinically silent access-site complications compared with assessment restricted to symptomatic patients.

### Limitations

The principal limitation of the study is that the two devices were used in completely non-overlapping periods. Therefore, device effects cannot be completely separated from period effects. The study cannot demonstrate the superiority of either device. In addition, the study had a retrospective, single-center design, and during the study period a VCD was the routine closure method for 6F transfemoral neurointerventional procedures; manual compression was used in selected cases, and these patients, together with those using non-6F sheaths, were excluded from the cohort according to the predefined exclusion criteria. Thus, there was no manual-compression control group in the study, and the findings cannot be interpreted as evidence that VCDs are superior to manual compression.

The limited sample size may have resulted in wide confidence intervals and borderline *p* values in some analyses. The inability to match 43 Obtura patients after propensity-score matching and the presence of residual imbalances at |SMD| > 0.10 for some covariates limit the generalizability of the matched analyses and leave open the possibility of residual confounding.

In addition, early ambulation and patient comfort could not be evaluated because all patients underwent standardized 4-h bed rest.

Finally, not all potential confounders, including body mass index (BMI), femoral artery diameter, number of puncture attempts, anatomical characteristics of the puncture site, procedure duration, duration of sheath dwell within the artery, and serial ACT measurements, were reliably available in the retrospective dataset. Therefore, the observed associations may not be completely free from unmeasured or residual confounding. The findings should be considered hypothesis-generating within this context.

Patients without a 30-day Doppler examination were excluded from the study; because their access-site outcomes could not be assessed and they may have differed from the analyzed cohort, a degree of selection bias cannot be excluded.

These limitations directly inform the design of future studies. We recommend that future studies compare the devices concurrently. A randomized trial would be ideal; if this is not feasible, randomization by alternating time blocks could be used. A multicenter design should be preferred to improve generalizability.

Assuming technical success of 52.9% with StarClose, a two-sided α of 0.05, and 80% power, approximately 121 patients per group (242 in total) would be required to detect the crude difference observed in our study (70.4% with Obtura). Because this difference may have been inflated by the period effect, a more conservative estimate based on the effect observed after propensity-score matching (OR, 1.55; corresponding to 63.6% with Obtura) would require approximately 338 patients per group (676 in total), or approximately 752 patients in total allowing for 10% attrition. Safety comparisons would require substantially larger samples: detecting a difference in access-site complications of 1% vs. 3% with 80% power would require approximately 768 patients per group. Variables that were not reliably available in our retrospective dataset and should be collected prospectively include body mass index; systolic and diastolic blood pressure before and at closure; the type and intensity of antithrombotic therapy; baseline hemoglobin and platelet count; femoral artery diameter, graded calcification, and sheath-to-femoral artery ratio; side, level, and angle of puncture; number of puncture attempts and puncture depth; procedure duration and sheath dwell time; sheath size and sheath exchange; serial ACT measurements; weight-based heparin dose and protamine reversal; operator experience; and device–sheath compatibility.

## 5. Conclusions

In this single-center retrospective study, technical success was 52.9% in the StarClose period (2022–2024) and 70.4% in the Obtura period (2025). Because the two devices were used in completely non-overlapping periods, device effects cannot be separated from period effects. Moreover, the difference was not statistically significant after multivariable adjustment or propensity-score matching, and its direction reversed when a ≤7-min hemostasis threshold was applied. The present data are therefore insufficient to demonstrate the superiority of either device. The shorter rescue compression after StarClose failure and the low access-site complication rates observed with both devices should be interpreted descriptively, given the retrospective, non-standardized records and the small number of events.

The main contribution of this study is to provide a basis for the design of future research. Prospective, concurrent, adequately powered comparative studies with standardized endpoints are needed to determine whether the observed differences reflect true device effects.

## Figures and Tables

**Figure 1:**
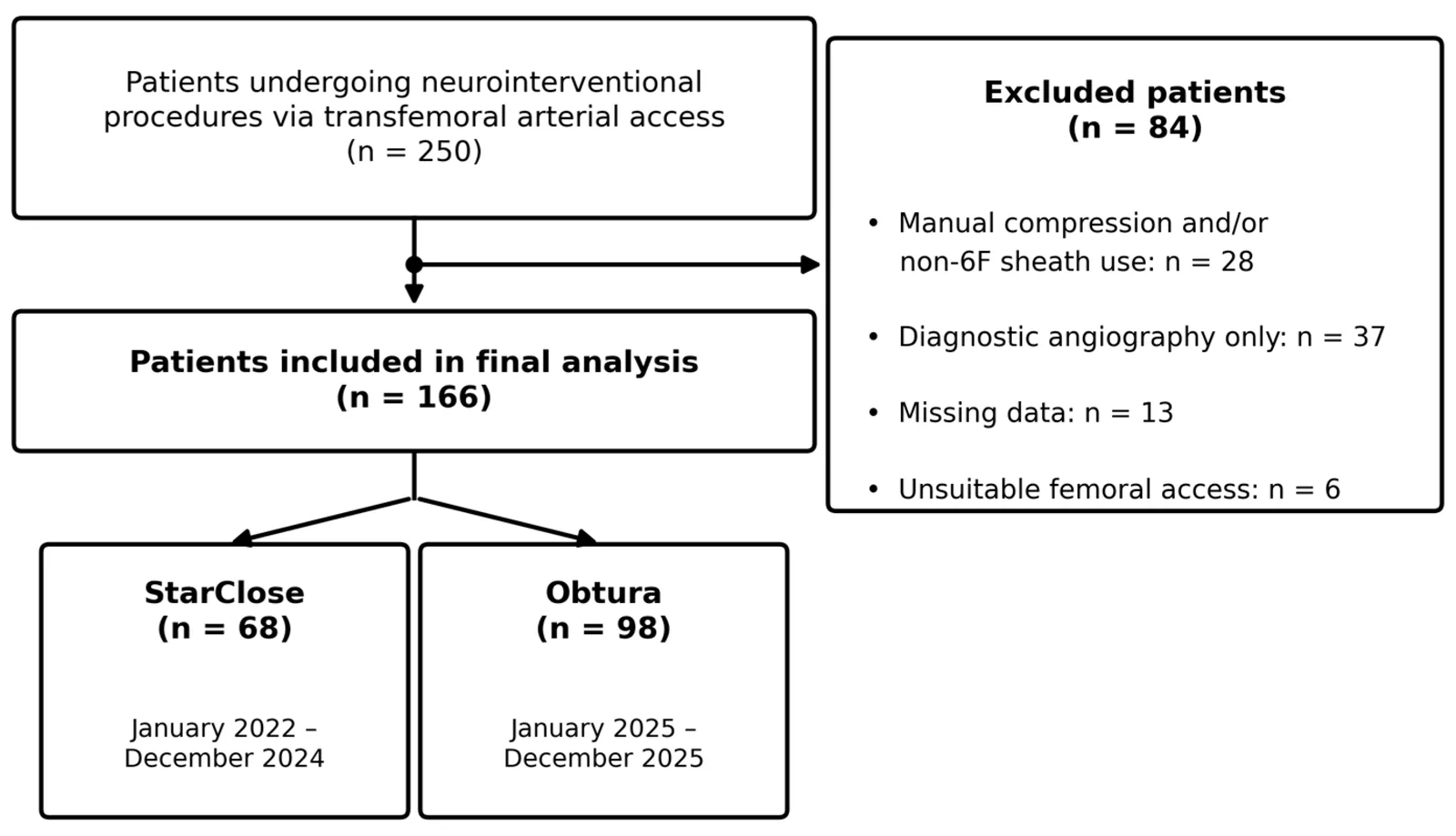
Patient flow diagram. Patients were treated in two consecutive, non-overlapping periods (StarClose, January 2022–December 2024; Obtura, January–December 2025).

**Table 1:** Baseline demographic and clinical characteristics by device.

Variable	StarClose (*n* = 68)	Obtura (*n* = 98)	*p* Value	SMD
Age, years, mean ± SD	64.0 ± 12.6	66.1 ± 12.2	0.217	−0.17
Female sex, %	32.4	43.9	0.148	−0.24
Diabetes mellitus, %	29.4	36.7	0.404	−0.16
Hypertension, %	58.8	57.1	0.874	0.03
Smoking, %	38.2	38.8	1.000	−0.01
Systolic BP, mmHg, mean ± SD	118.4 ± 22.3	122.6 ± 17.2	0.060	−0.21
Diastolic BP, mmHg, mean ± SD	65.2 ± 10.6	66.6 ± 9.8	0.363	−0.14
Aspirin use, %	33.8	53.1	**0.017**	−0.40
Clopidogrel use, %	23.5	37.8	0.063	−0.31
Enoxaparin use, %	2.9	6.1	0.473	−0.15
Composite antithrombotic burden score, median (IQR)	0 (0–1)	1 (0–2)	**0.011**	—
Sheath size, F	6.0 (all patients)	6.0 (all patients)	— *	— *
Intraprocedural heparin use, %	100.0	100.0	—	—
Heparin dose, IU, mean ± SD	5117.6 ± 560.6	5040.8 ± 198.9	0.232	0.18

SMD: standardized mean difference; |SMD| > 0.10 was considered a possible imbalance. Bold *p* values indicate statistical significance (*p* < 0.05). IQR: interquartile range. SD: standard deviation; BP: blood pressure. Composite antithrombotic burden score: sum of aspirin, clopidogrel, and enoxaparin use before the procedure (range 0–3). * Sheath size was 6F in all patients (an inclusion criterion); therefore, no comparison was performed.

**Table 2:** Distribution of procedure type.

Procedure Type	StarClose (*n* = 68)	Obtura (*n* = 98)
Aneurysm embolization (coil/stent/flow diverter)	4 (5.9%)	5 (5.1%)
Carotid/intracranial stenting	15 (22.1%)	33 (33.7%)
Mechanical thrombectomy	49 (72.1%)	59 (60.2%)
Other	0 (0%)	1 (1.0%)

Fisher’s exact test, *p* = 0.285 (across all 4 categories).

**Table 3:** Multivariable logistic regression model (dependent variable: technical hemostasis success; *n* = 166).

Variable	OR	95% CI	*p*
Device (Obtura vs. StarClose)	1.99	0.99–3.99	0.053
Age (per year)	1.02	1.00–1.05	0.089
Female sex (vs. male)	0.70	0.35–1.42	0.326
Aspirin use	1.78	0.33–9.60	0.502
Composite antithrombotic burden score (per point)	0.65	0.20–2.05	0.459
Mechanical thrombectomy (vs. other procedures)	0.23	0.07–0.77	**0.017**

OR, odds ratio; CI, confidence interval (Wald method). The composite antithrombotic burden score was calculated as the sum of three binary indicators of baseline antithrombotic therapy before the procedure: aspirin (0 = no, 1 = yes), clopidogrel (0/1), and enoxaparin (0/1), giving a range of 0–3; intraprocedural heparin, which was administered to all patients, was not included. The score was entered as a continuous variable (OR per 1-point increase); a likelihood-ratio test comparing continuous and categorical (0, 1, ≥2) specifications showed no evidence of non-linearity (*p* = 0.83). Because aspirin is also a component of the score, a sensitivity analysis excluding aspirin from the model yielded a similar device estimate (OR, 1.99; 95% CI, 0.99–4.00). Model performance: EPV = 10.2 (61 events/6 predictors); c-statistic = 0.683; Hosmer–Lemeshow χ^2^ = 11.63 (df = 8), *p* = 0.168. Bold *p* values indicate statistical significance (*p* < 0.05).

**Table 4:** Covariate balance after matching (StarClose *n* = 55, Obtura *n* = 55).

Variable	StarClose	Obtura	SMD (Post-Matching)	SMD (Pre-Matching)
Age, years, mean	64.9	64.0	0.07	−0.17
Female sex, %	38.2	45.5	−0.15	−0.24
Diabetes mellitus, %	32.7	29.1	0.08	−0.16
Hypertension, %	58.2	56.4	0.04	0.03
Smoking, %	36.4	43.6	−0.15	−0.01
Systolic BP, mmHg, mean	121.0	121.9	−0.04	−0.21
Diastolic BP, mmHg, mean	66.4	65.5	0.08	−0.14
Aspirin use, %	38.2	34.5	0.08	−0.40
Clopidogrel use, %	29.1	23.6	0.12	−0.31
Enoxaparin use, %	3.6	1.8	0.11	−0.15
Heparin dose, IU, mean	5090.9	5054.5	0.08	0.18
Procedure type (3 categories), SMD range	—	—	−0.04 to 0.07	approximately −0.3 to 0.3

SMD: standardized mean difference. The pre-matching column corresponds to the SMDs for the full cohort in Table 1.

**Table 5:** Sensitivity analysis of the success threshold definition (unadjusted and adjusted comparisons).

Total Threshold (Additional Compression)	StarClose Success, *n*/*N* (%)	Obtura Success, *n*/*N* (%)	Crude OR	95% CI	*p*	Adjusted OR	95% CI	*p*
≤2 min (primary; gentle pressure only)	36/68 (52.9)	69/98 (70.4)	2.11	1.11–4.03	**0.033**	1.99	0.99–3.99	0.053
≤3 min (additional ≤1 min)	36/68 (52.9)	69/98 (70.4)	2.11	1.11–4.03	**0.033**	1.99	0.99–3.99	0.053
≤5 min (additional ≤3 min)	37/68 (54.4)	70/98 (71.4)	2.09	1.10–4.00	**0.032**	1.98	0.99–3.96	0.055
≤7 min (additional ≤5 min)	52/68 (76.5)	70/98 (71.4)	0.77	0.38–1.57	0.592	0.64	0.29–1.39	0.257

OR: odds ratio for success with Obtura relative to StarClose; crude *p* values from Fisher’s exact test; CIs by the Wald method. Adjusted ORs are from the multivariable model in Table 3 (age, sex, aspirin use, composite antithrombotic burden score, and mechanical thrombectomy). Bold *p* values indicate statistical significance (*p* < 0.05).

**Table 6:** Additional manual compression time in technical failures.

Metric	StarClose (*n* = 32)	Obtura (*n* = 29)	*p* *
Median, min (IQR)	5.5 (5.0–6.25)	10.0 (8–14)	**4.8 × 10^−7^**
Median excluding outliers, min	5.0	10.0	**1.3 × 10^−7^**

* Mann–Whitney U test. IQR: interquartile range. Outliers: StarClose 35 min (ACT 330 s), Obtura 30 min (ACT 320 s). Bold *p* values indicate statistical significance (*p* < 0.05).

**Table 7:** Comparison of vascular complications between two groups.

Complication *n* (%)	StarClose (*n* = 68)	Exact 95% CI	Obtura (*n* = 98)	Exact 95% CI	*p*
Pseudoaneurysm	1 (1.5%)	0.0–7.9	1 (1.0%)	0.0–5.6	1.000
Hematoma	1 (1.5%)	0.0–7.9	1 (1.0%)	0.0–5.6	1.000
Major bleeding (whole cohort, *n* = 166)	0/166 (0%)	0.0–2.2	—	—	—

CI: confidence interval (exact binomial method). The pseudoaneurysm and hematoma in the Obtura group were recorded in the same patient.

**Table 8:** Technical success and access-site complications in comparative and device-specific VCD studies and in the present study.

Study	Design, Population, Sheath	Devices (*n*)	Technical Success	Access-Site Complications
Schulz-Schüpke et al., 2014 (ISAR-CLOSURE) [14]	RCT; coronary angiography; 6F	FemoSeal (1509) vs. ExoSeal (1506)	Device failure 5.3% vs. 12.2%	30-day composite ^a^: 6.0% vs. 7.8%
Kim et al., 2019 [15]	Prospective, non-randomized; PCI; mainly 6F	StarClose (200) vs. Angio-Seal (200)	StarClose deployment failure or insufficient closure: 16 patients ^b^	Overall bleeding 43.5% vs. 56.0%; complicated access-site bleeding 0.5% vs. 5.0%
Gonen et al., 2021 [17]	Retrospective; complex endovascular procedures under intensive anticoagulation	Angio-Seal vs. StarClose (166 in total)	Deployment success 100% with both devices	No major complications; minor complications similar
Lai et al., 2024 [16]	Meta-analysis; 9 studies; 5466 patients	Angio-Seal vs. StarClose	Closure success RR 1.05 (95% CI, 1.00–1.09)	Overall complications RR 0.61 (95% CI, 0.29–1.29)
Himanshu et al., 2026 [12]	RCT, four arms; mainly PCI	Angio-Seal (250) vs. Obtura (250) vs. ProGlide (250) ^c^	99.1% vs. 97.4% vs. 94.6% ^d^	Hematoma 0.8% vs. 2.0% vs. 3.2%; pseudoaneurysm 0% vs. 0.8% vs. 0.8%
Padhi et al., 2026 [13]	Retrospective, single device; neurointerventional	Obtura 8F (100)	Hemostasis success 96%	Hematoma or pseudoaneurysm 6%
Present study	Retrospective, non-concurrent periods; neurointerventional; 6F	StarClose (68) vs. Obtura (98)	52.9% vs. 70.4% ^e^	Any access-site complication ^f^ 2.9% vs. 1.0%; major bleeding 0%

RCT, randomized controlled trial; PCI, percutaneous coronary intervention; RR, risk ratio; CI, confidence interval; VCD, vascular closure device. Values are presented in the order in which the devices are listed. ^a^ Composite of vascular access-site complications at 30 days. ^b^ Patients with failed StarClose deployment were excluded from that study’s outcome analysis; no Angio-Seal failures were reported. ^c^ A manual compression arm (*n* = 250) was also included. ^d^ Up to 3 min of light compression was permitted. ^e^ Hemostasis within 2 min of deployment, allowing gentle manual pressure during this interval; the two devices were used in non-overlapping periods. ^f^ Patient-level rate (pseudoaneurysm or hematoma). Definitions of technical success and complications, populations, and sheath sizes differ across studies; absolute rates are therefore not directly comparable.

## Data Availability

The datasets generated and/or analyzed during the current study are not publicly available because they contain patient-related clinical information and are subject to institutional and privacy restrictions. De-identified data may be made available from the corresponding author upon reasonable request and subject to institutional approval and applicable data-protection regulations.

## Supplementary Materials

The following supporting information can be downloaded at https://www.mdpi.com/article/10.3390/jcm15218242/s1. Table S1: Technical success by device within procedure-type subgroups and test for device × procedure interaction; Table S2: Technical success and additional manual compression by device period in exploratory clinical scenarios.

## Abbreviations

ACT, activated clotting time; BARC, Bleeding Academic Research Consortium; BMI, body mass index; BP, blood pressure; CI, confidence interval; EPV, events per variable; F, French; IQR, interquartile range; OR, odds ratio; PCI, percutaneous coronary intervention; PS, propensity score; PSM, propensity-score matching; RCT, randomized controlled trial; RR, risk ratio; SD, standard deviation; SMD, standardized mean difference; VCD, vascular closure device.
