# Supplementary material for: Comparison of the Technical Success of Obtura and StarClose Systems for Femoral Arterial Access-Site Closure in Neurointerventional Procedures: A Single-Center Retrospective Cohort Study
Source: J Clin Med. 2026 Oct 23;15(21):8242. doi: 10.3390/jcm15218242 (PMC13764310; doi:10.3390/jcm15218242)
Supplement: Supplementary file 1 [file jcm-15-08242-s001.zip › jcm-4605567-supplementary.pdf]

Supplementary Materials

Comparison of the Technical Success of Obtura and StarClose Systems for Femoral Arterial Access-Site Closure in Neurointerventional Procedures: A Single-Center Retrospective Cohort Study

Nurcan Kat and Tirdad Setayeshi

Table S1. Technical success by device within procedure-type subgroups and test for device × procedure interaction.

Subgroup	StarClose n/N (%)	Obtura n/N (%)	Crude OR (95% CI)	*p*	Adjusted OR [a] (95% CI)	*p*
Mechanical thrombectomy (n = 108)	24/49 (49.0)	35/59 (59.3)	1.52 (0.71–3.26)	0.334	1.58 (0.70–3.59)	0.273
Other procedures [b] (n = 58)	12/19 (63.2)	34/39 (87.2)	3.97 (1.06–14.89)	0.045	4.34 (1.07–17.53)	0.039
Device × mechanical thrombectomy interaction [c]	—	—	—	—	0.37 (0.08–1.78)	0.216

OR, odds ratio for technical success with Obtura relative to StarClose; CI, confidence interval. Crude *p* values from Fisher's exact test; ORs with Wald 95% CIs. [a] Adjusted for age, sex, aspirin use, and composite antithrombotic burden score. [b] Aneurysm embolization (n = 9), carotid/intracranial stenting (n = 48), and other (n = 1). [c] Interaction term added to the multivariable model in Table 3 (device, age, sex, aspirin use, composite antithrombotic burden score, and mechanical thrombectomy); likelihood-ratio test $p = 0.213$. With Firth penalized logistic regression, adjusted ORs were 1.55 (95% CI, 0.68–3.51) in the mechanical thrombectomy subgroup and 3.66 (95% CI, 0.97–13.87; $p = 0.056$) in the other-procedure subgroup.

Table S2. Technical success and additional manual compression by device period in exploratory clinical scenarios.

Scenario	StarClose success, n/N (%)	Obtura success, n/N (%)	*p*[a]	Rescue compression[b], median, min StarClose / Obtura	Additional compression per patient[c], mean, min StarClose / Obtura
All patients	36/68 (52.9)	69/98 (70.4)	0.033	5.5 / 10.0	3.1 / 3.3[d]
Procedure type[e]					
Emergency mechanical thrombectomy	24/49 (49.0)	35/59 (59.3)	0.334	6.0 / 10.0	3.6 / 4.5
Elective carotid/intracranial stenting	8/15 (53.3)	30/33 (90.9)	0.006	5.0 / 15.0	2.5 / 1.4
Aneurysm embolization	4/4 (100)	4/5 (80.0)	1.000	— / 6.0	0.0 / 1.2
Composite antithrombotic burden score[f]					
0	22/43 (51.2)	24/42 (57.1)	0.665	6.0 / 10.0	2.9 / 4.6
≥1	14/25 (56.0)	45/56 (80.4)	0.031	5.0 / 14.0	3.5 / 2.3
≥2	9/16 (56.2)	32/37 (86.5)	0.029	5.0 / 15.0	2.3 / 1.7

All analyses are exploratory and unadjusted; subgroups overlap, and the two devices were used in non-overlapping periods (StarClose, 2022–2024; Obtura, 2025). [a] Fisher's exact test for technical success. [b] Additional manual compression among patients with technical failure. [c] Additional manual compression averaged over all patients in the subgroup, counting 0 min for patients with technical success. [d] Mann–Whitney U test, $p = 0.37$. [e] One patient with another procedure type (Obtura period) is not shown separately. Device × mechanical thrombectomy interaction in the multivariable model: $p = 0.216$. [f] Sum of aspirin, clopidogrel, and enoxaparin use (range 0–3). Device × antithrombotic burden interaction: $p = 0.054$ (continuous score) and $p = 0.064$ (score ≥1 vs. 0).
